# ‘I was in need of somewhere to release my hurt:’ Addressing the mental health of vulnerable adolescent mothers in Harare, Zimbabwe, through self-help groups

**DOI:** 10.1080/16549716.2022.2040151

**Published:** 2022-03-24

**Authors:** Rudo Chingono, Constance Kasese, Sam Miles, Joanna Busza

**Affiliations:** a Centre for Sexual Health and HIV/AIDS Research, Harare, Zimbabwe; b Biomedical Research and Training Institute, Harare, Zimbabwe; c Institute of Global Health, University College London, UK; d Department of Public Health, Environment & Society, London School of Hygiene and Tropical Medicine, London, UK

**Keywords:** Adolescence, community programmes, intervention, mental health, qualitative methods

## Abstract

**Background:**

Adolescents experiencing multiple vulnerabilities, including poverty, curtailed education, transactional sex and early childbearing, are at risk of poor mental health. In Zimbabwe, girls who are pregnant or new mothers and involved in selling sex struggle to cope with the combined pressures of parenthood, financial insecurity, and social stigma. A pilot intervention brought such girls together into self-help groups to increase peer support, resources and skills.

**Objective:**

This study aimed to explore whether and how participation in a self-help group intervention affected vulnerable young mothers’ experiences and perceptions of mental health stressors.

**Methods:**

Self-help groups received 12 participatory sessions over 6 months. Eighteen semi-structured interviews and three focus group discussions were held with participants and drop-outs. Before and after the intervention, participants completed the locally validated 14-item Shona Symptom Questionnaire tool to indicate the probable prevalence of common mental health disorders.

**Results:**

Adolescent girls described mutually reinforcing stressors in their lives and reported low self-esteem and anxiety. Key themes emerging from qualitative data centred around girls’ struggles with adverse life events, the burden of new motherhood, social isolation related to sex work and self-help groups as a source of hope. Participants joined groups to obtain support and felt their mental well-being improved due to new social networks, feelings of solidarity with peers, and increased confidence for positive action, that is, seeking health services for themselves and their babies. Prior to enrolment 16% showed signs of possible common mental disorders falling to 2% at follow-up.

**Conclusions:**

Participants believed involvement in interactive self-help groups improved their mental health by strengthening peer support and engendering hope for the future. Although reduced mental distress cannot be attributed to the programme, the pilot intervention offers a low-cost approach that could be rigorously tested and adapted to a wide range of community settings.

## Background

The mental health needs of adolescent girls and young women (AGYW) are known to intersect with other health vulnerabilities, including unintended pregnancy and early childbearing [1]. Compared to adult mothers, AGYW experience higher prevalence of depression, anxiety and other common mental health disorders during pregnancy and the post-partum period [2]. The combined pressures of caring for a new baby, struggling financially, and experiencing societal discrimination can lead to significant stress and anxiety [3]. Feelings of isolation and a lack of social support can further exacerbate maternal depression [4].

In sub-Saharan Africa, sexually active AGYWs are also at disproportionate risk of HIV, which puts additional pressures on their mental health [5,6]. Among the most vulnerable to HIV are AGYW involved in transacting or selling sex [7–9]. Global estimates suggest that 20–40% of female sex workers initiate sex work before the age of 18, yet AGYW who sell sex are often excluded from both research and services [10–12] despite facing well-documented risks to their physical, sexual and mental health [13–17]. Compared to older peers, young women report poorer capacity to negotiate condom use, higher rates of violence from sexual partners, and lower uptake of preventive and treatment services for HIV and other sexual and reproductive health needs [18].

In Zimbabwe, high numbers of AGYW experience both early childbearing and HIV risk, many as a result of their involvement in exchanging sex for money or material benefits [19,20]. Nationally, 21.6% of all girls aged 15–19 have begun childbearing, and this is higher among those in the lowest wealth quintile (33.6%) and with primary or lower education (37.8%); by age 25, 91% of the women have given birth at least once [21]. Education levels remain low, and for many adolescents and young mothers, transacting sex is one of the few livelihood options to provide for themselves and their children, yet this places them at high risk of HIV and other adverse health outcomes. Among sex workers, HIV prevalence rises sharply from 20.7% of 18–19 year-old sex workers to 38% among the 20–24 age group [22]. Research into postpartum depression among adolescent mothers (<18) in Zimbabwe found double the prevalence compared to adult mothers (13% vs 7%) [23].

A few evidence-based interventions have addressed adolescent mental health in resource-poor settings [24] particularly in the context of AGYW experiencing multiple vulnerabilities [6,25]. To help fill this gap, the *Building Resilience through Self-Help Groups* (SHG) intervention was introduced in Harare, Zimbabwe, specifically tailored to AGYW involved in selling sex who were pregnant, new mothers or both. Due to known higher behavioural risk in this context among AGYW who consider themselves to be sex workers rather than engaged in informal transactional sex [7], the intervention focused on girls who self-reported that selling sex was their primary means of financial support. The aims of the intervention were to reduce feelings of isolation, build social support networks, and improve participants’ life skills including parenting, financial literacy and self-care in the context of facilitated peer groups. The rationale for interactive self-help groups was that bringing pregnant and new mother adolescent sex workers together would provide a platform for psychosocial support that would lead to improved self-confidence, uptake of clinical services and reduced risk of common mental health disorders. This paper examines participants’ mental health stressors and how these may have been influenced through participation in the SHG intervention.

## Methods

### Study setting and population

The SHG intervention was delivered as part of Zimbabwe’s nationally scaled HIV prevention and treatment programme for sex workers *Sisters with a Voice (Sisters)*, which offers a wide range of social and clinical services for high-risk women, including AGYW [19,22,26–29]. The intervention was delivered in four high-density communities in Harare, Zimbabwe, characterised by small, clustered houses shared by several families, low education levels of resident adults, low-wage and informal employment, and high turnover of residents. These communities were selected because they were known as venues where sex is sold and because they had active *Sisters* programme peer educators who could assist with recruiting participants. Eligible participants were adolescent girls aged 16–19 years, residing in the selected communities, who reported selling sex as their main means of earning a livelihood, and were either currently pregnant or had at least one child up to 24 months old. Screening was conducted by *Sisters* peer educators, who identified AGYW through their personal networks, approached potential participants, gauged their interest, and referred them to a dedicated project facilitator.

### Intervention and procedures

Formative work was conducted over 1 month to assess acceptability and understand AGYW’s preferred design and content of self-help groups to ensure the intervention met local needs and preferences. A package of 12 participatory sessions delivered over 6 months was developed, including a facilitator’s manual and participant handbook to guide the sessions. A trained facilitator (second author) coordinated all the sessions to ensure consistency and quality of sessions across the groups. Early activities aimed to build rapport and trust within the group and progressed to developing individual and group problem-solving skills, practice-based learning for healthy parenting and safer sexual practices, the importance of self-care and appropriate health-seeking, and improving capacity to manage and save money. Throughout the SHG program, the uptake of clinical and psychosocial support services offered by *Sisters* was encouraged by staff, who also referred participants to other services, such as subsidised education and vocational training.

The intervention was conducted between February and September 2019. Seven SHGs were established. Each had 10–15 participants recruited by *Sisters* peer educators who knew adolescent girls within their communities who sell sex. The SHG intervention was conducted as a pilot project to assess the feasibility and acceptability of engaging a group of highly vulnerable AGYW with experience of selling sex, pregnancy and parenthood.

### Data Collection and Analysis

The intervention was not primarily intended as research nor conducted under trial conditions. However, to document the process, routine programme data was collected by the second author on attendance rates, number and frequency of sessions, and facilitated referrals to health and social services.

To better understand the acceptability of self-help group interventions, 18 semi-structured interviews were conducted with participants at 4 and 6 months, with a target of interviewing 1–2 AGYW per self-help group. Three focus group discussions (FGDs) were conducted, in 3 out of 4 programme communities near the end of implementation. FGDs were used to build rapport among AGYW drawn from different SHG and who did not necessarily know each other. Discussion topics addressed the structure, content, and process of the SHG intervention without asking about personal experiences. Interview participants were purposively selected to include both active participants and drop-outs. Semi-structured topic guides were included with questions on participants’ motivations for joining SHG and perceptions of the intervention and its effects. These were conducted by the first author, a trained female social scientist affiliated with the wider *Sisters* programme but not involved with facilitation of the SHG. All interviews and focus groups were conducted in the local language (Shona) and audio recorded. Interviews were transcribed, translated into English by the second author, before being anonymised, re-read and discussed during a period of familiarisation by all researchers. Detailed notes on the main topics and areas of agreement and disagreement were used for the analysis of FGDs, which were not transcribed, but served as background information on the general implementation of the SHG from participants’ perspectives. The first author manually developed a content analysis coding framework combining inductive and deductive themes (Table 1). Inductive codes emerged directly from narratives, for example, ‘seeking support’, while deductive codes examined programme-related motivators, facilitators and barriers. The framework was applied across all interview transcripts and the principles of narrative analysis were used to compare AGYWs’ accounts as holistic representations of their subjective experience of the intervention and its effects on their mental health [30].

**Table 1.** Key themes and codes

Theme	Codes
Struggles with life stressors	Difficult childhood & life experiences
	Failed relationships: rejection, abuse
	Low sense of self-worth
	Lack of social support
	Need for mental health support
The burden of motherhood	Financial difficulties and instability
	Forced maturity
	Inability to provide for child
	Children as a source of comfort
Social stigma and isolation related to sex work	Healthcare worker attitudes
SHG as a source of hope	Empowerment
	Enhanced parenting skills
	Future aspirations
	Increased social networks/companionship
	Improved mental well-being
Experience of SHG	Reasons for joining/Motivation
	Accessibility/ability to attend
	Reasons for drop out – lack of interest
	Barriers to attendance
Perceptions of SHG experience	Positive perceptions
	Negative perceptions
	Suggestions for improvements
	Content
	Process (timing, group size, meeting frequency)

Finally, short questionnaires were administered to all SHG participants before and after the 12 participatory sessions that elicited socio-demographic data, perceived practical and emotional support from peers, financial literacy, health service use, and included the locally validated Shona Symptom Questionnaire (SSQ-14) to indicate the probable prevalence of common mental health disorders [31]. Basic frequencies and cross tabulations were calculated to better understand participants’ backgrounds, needs, and experiences of the intervention. This quantitative tool was employed as a means to monitor the likely prevalence of common mental health disorders (CMD) over time but not to measure attributable changes given the lack of comparison sites.

Ethical approval was obtained from the Medical Research Council of Zimbabwe (MRCZ/A/2370) and London School of Hygiene and Tropical Medicine (LSHTM/15920) and the programme was registered with the Research Council of Zimbabwe. Written informed consent was sought from all participants using forms developed, piloted and tailored to participants’ age and education levels.

## Results

In total, 93 AGYW joined SHG, of whom all participated in the baseline survey, and 54.8% (n = 51) completed the follow-up survey. Among participants, 25 were 16–17 years old, and the rest 18 or 19. Twenty reported having no or only primary-level schooling, while the rest had attended some secondary education, but none had completed school nor were enrolled at the time of joining an SHG. After six months of SHG implementation, 6 out of 7 groups remained, each with 9–13 active participants. Over the course of the intervention, 28 AGYW dropped out (30.1%) and 14 were referred into an educational support programme and therefore no longer attended the SHG due to their school commitments. Among those who dropped out, 13 did so between sessions 2–3, 10 dropped out between sessions 4 and 6, and 5 between sessions 7–9. A high number, 81.8% (n = 9), of participants dropped out from the 7^th^ group and the remaining participants were integrated into another group within the same community. AGYW reported relocation as the main reason for dropping out, although a few cited competing demands on their time and lack of interest. The final 6 groups completed all 12 sessions with 72% (n = 67) of these members attending half or more sessions.

During interviews and FGDs, participants described multiple and intersecting sources of stress and anxiety in their lives, ranging from difficult childhoods and family experiences, to the challenges they confronted as new mothers reliant on sex work to support themselves and their babies. They described the desire to join self-help groups to combat feelings of isolation and reported positive experiences in which participating in such support groups relieved their distress.

Qualitative findings can thus be categorised into the following key themes: struggles with life stressors; the burden of motherhood; social stigma and isolation related to sex work; and SHG as a source of hope. These themes are detailed in the sections below, all excerpts are from individual interviews.

### Struggles with life stressors

Interviewees described how difficult life circumstances led to feelings of depression and anxiety. For some, abuse within the household pushed them to leave home at a young age and they began to sell sex to survive. Others reported that their lives took a negative turn when they found themselves in a relationship in which they experienced verbal, emotional and/or physical abuse. What these stories have in common is AGYW’s belief that they had ended up as single mothers who relied on selling sex due to circumstances beyond their control. AGYW saw their life choices as reactions to a series of adverse events, leading to a certain resigned acceptance of their current circumstances:

I met a guy whom I started dating. The relationship went well up until I got pregnant and eloped to his parents’ house […] only to discover that he had a wife […] I was verbally and emotionally abused […] I saw it best to leave him and go back to selling sex and taking care of myself and my pregnancy […] that has been my life since then. (Community 2, aged 21)

Failed relationships invariably caused emotional distress, which was exacerbated during AGYWs’ pregnancies. Several respondents had initially hoped their pregnancy might lead to greater commitment and permanence of their relationships. While participants had not undergone formal or legal marriage, they considered themselves to be married once they were expecting a child. As described below, the disintegration of relationships under these circumstances could be deeply hurtful:

My major challenge when I was pregnant was my husband leaving me. That was the most stressful phase and it took time for me to get over it. (Community 3, aged 18)

### Burden of motherhood

Failure in one area of personal life negatively affected AGYW’s confidence in their ability to cope with becoming young mothers. While support from others could have reduced these feelings of inadequacy at a sensitive time, respondents emphasised that they were lacking social networks through which they could obtain emotional or practical assistance. The previous interviewee linked the lack of support network at such a difficult time to her feelings of inadequacy as a parent:

Older mothers are better equipped to take care of their children. They are likely to have money […] or someone to provide for them. Others have parents, aunts, and friends but I do not. (Community 3, aged 18).

Financial support was a particular concern for AGYW. While participants experienced financial concerns throughout their lives, such as losing parents or leaving school due to inability to pay fees, they found not being able to turn to others for help with their parenting responsibilities acutely upsetting:

Being unable to provide for my child stresses me. The days when I struggle to make enough money I feel like crying. I just want to be able to provide for my child, and, being the sole provider, parenting can be difficult (Community 4, aged 19)

The times that have been frustrating are those when my child got sick and I needed money to buy medication and did not have any. Knowing that even if I called the father it would be pointless is even more frustrating as in times like those that is when I wish I was not a single mother and I had someone else to help fend for the child. (Community 2, aged 21).

### Social Stigma and Isolation Related to Sex Work

Involvement in selling sex depreciated AGYW self-esteem, particularly in the face of stigma in the community, compounded by their status as single mothers. The resulting isolation was both identified and acknowledged by participants, most of whom openly stated feeling in need of mental health support. Reducing isolation was one of the main drivers for joining an SHG, and was perceived as so valuable a resource that at least 2 participants admitted they had falsely claimed they were pregnant in order to be eligible for the program:

I got into this program in the pretence of being pregnant as I wanted to gain access […] to counselling services […]. I was in need of somewhere to release my hurt. I wanted to be able to talk and not be judged about my past. I am still in pain and now I get to work through that pain […] A lot of us have been through stressful events, we need help and this program through its linkage to counselling will help us work on the stressful life experiences. (Community 4, aged 19)

The participant went on to explain that while counselling services were available at the local public health facilities, she did ‘not feel comfortable going to ‘open up’ about problems to the local nurses’ because ‘most of them are old and they are judgemental’. This reflects wider fears about stigmatising attitudes towards AGYW perceived to contravene sexual mores and acceptable behaviour. Fearing unfriendly service providers, AGYW minimised engagement with any health or social services, including pregnancy care or HIV testing and treatment. For example, one participant stopped attending a clinic for family planning after being scolded by the nurse.

The nurse said ‘go to the end of the line, why do you rush to get pregnant and be sexually active as young girls?’ […] I have now stopped asking for pills there, I would rather buy them at a tuckshop or at the pharmacy. (Community 2, aged 18)

Other social stigmas included rejection from family members, accusations of ‘stealing husbands’ from neighbours and belief that they deserved bad outcomes as a result of their ‘promiscuity’ (e.g. for contracting HIV).

### Self-Help Groups as a Source of Hope

Joining an SHG represented an opportunity to enter a socially supportive space, engaging with peers and friendly, non-judgmental staff. Participants who had little experience of formal education also perceived the intervention as an alternative learning environment and enrolled as a means to obtain new knowledge and skills.

I decided to join because I do not have other people to teach me. I wanted to learn about parenting. I wanted to be advised like everyone else. I do not have people to support me and I hoped this group would fulfil that desire of mine. (Community 4, aged 22)

Interviewees also found that the interactive sessions alleviated stress by encouraging them to share difficult experiences and work together to address problems. While the programme facilitator was appreciated as a source of information and mentoring, AGYW focused on their interactions with peers, and resulting feelings of reduced isolation. Over the course of the workshops, trust deepened, genuine friendships were formed, and participants met up outside of the organised meetings.

At first people did not interact beyond what they had to do in the sessions. One would come to the session, after it was finished, one would get refreshments and leave. No one really made the effort of getting to know the other person. This changed as the sessions went on. We started to trust each other more, open up more and slowly friendships were developed and by the end of it people started spending time together outside of the session (Community 1, aged 22)

Participants credited SHG with helping reduce stress and reported that they were able to connect with others and develop a more positive outlook about the future. For some, envisioning some change toward a ‘better life’ or feeling able to view current circumstances through a different lens improved perceived mental health.

Being in a group has helped me meet someone I now consider my close friend … I am happy I at least have one friend who is encouraging me to be better in life […] not to think too much especially about my ex-husband leaving me (Community 3, 18 years old).

There are times I have come to the group stressed, then just being around people helped me stop thinking too much. At times someone even shares a problem they are going through and you realise that your situation is not as bad and you actually start having a fresh perspective to what you are going through (Community 4, aged 18)

Meeting other AGYW in similar circumstances not only helped participants feel less alone but gave them a sense of collective agency. Belief in the ability to work together with peers for shared benefit represented social capital that could be drawn on later:

Sex workers can be united when one of their counterparts is facing a challenge (Community 1, aged 19)

Access to tangible resources and opportunities, such as referrals into programmes that supported girls to get back into school, boosted self-confidence and helped counteract the low self-esteem they felt as sex workers:

I am glad I listened and that I came to the groups. It has paid off […] There is now hope in me [for] having a better life outside of sex work (Community 2, 21 years old)

### Shona symptom questionnaire results

The hope that emerged through qualitative analysis was reflected in the Shona Symptom scores measured prior to and after the intervention. Although the tool cannot diagnose mental health conditions, signs of common mental disorders reduced over time, with 16% (n = 15) of the young women screening positive of possible CMD at enrolment but only 2% (n = 1) screening positive at follow-up. At the initial baseline assessment 13% of the young women presented with low self-esteem with 0% reporting low self-esteem at the endline, and 10% of the participants at the baseline reported having high self-esteem, with this increasing to 16% at endline. On questions specific to involvement in the intervention, all participants who completed the endline survey reported that they felt more confident about themselves and that joining the SHG was useful to their lives, although those who had dropped out of the intervention did not fill out the endline questionnaire.

Those AGYW who dropped out of the intervention did not find SHG adequate to overcome their priority life challenges. For example, one young woman dropped out of the programme after her mother fell ill; at first, she stopped attending because she had travelled out of Harare to care for her mother, but she did not rejoin following her return. She blamed having dropped out on greater involvement in sex work, implying that emotional distress following her visit to her mother may have led to changes in sexual practices, as well as abandoning the programme.

I did not come back because most of the time I was either tired or nursing a hangover from a late night, or I had one of my long-term partners around […] I guess having gone [away] made me lose my morale (Community, aged 18 years)

## Discussion

This pilot project established self-help groups tailored to the needs of AGYW experiencing the intersecting vulnerabilities of being engaged in selling sex and experiencing early pregnancy and motherhood. During the intervention, indications of poor mental health were identified among participants resulting from a combination of factors including difficult childhood experiences, abusive relationships, financial instability, and stigma associated with selling sex to support themselves and their children. These emotional stressors coalesced into an increased risk of anxiety and depression and 16% of the participants presented with possible common mental health disorders at enrolment. The SHG created a platform for AGYW to develop a new network of social support in which they did not feel alone or judged for being young mothers or involved in selling sex. Many participants believed the intervention alleviated their mental distress, although any attributable effect on common mental health disorders could not be determined through this study.

As found in other research, young motherhood generated feelings of isolation, as did involvement in selling sex, and both were intertwined with poverty and deprivation, which are also known determinants of anxiety [1,5,32]. Selling sex was often a survival strategy following unintended pregnancy and relationship breakdown. Similar to other studies [33,34], the stigma experienced by AGYW related to selling sex and adolescent motherhood had negative effects on their mental health. Fear of judgmental attitudes also translated into avoidance of health services, which has been shown to have consequences for physical and sexual health [34,35].

Despite growing evidence that both AGYW who sell sex and those who are young mothers experience multiple mental health challenges, there have been few interventions that holistically address these [5,36,37]. This study found that introducing an opportunity to build support, reduce isolation and provide practical and material assistance helped meet many of AGYW’s needs related to their struggles to cope with looking after their children in a context of severe economic deprivation. Participants appeared to benefit most from the expansion of their social network and felt their stress and anxiety were alleviated through meeting and discussing shared experiences with peers. This resonates with findings from a recent systematic review of studies on the mental health of adolescents and young people living with HIV in which higher social support was the only factor significantly associated with lower rates of anxiety [38]. Peer-led social support also underpins successful interventions to improve vulnerable adolescents’ risk behaviour and engagement with services [39–41].

### Strengths and limitations

A strength of the intervention is that it is replicable and adaptable to different contexts, relying on a mix of trained facilitation and peer-led participation to identify and discuss locally salient life stressors like poverty, early marriage, abuse, pregnancy and parenting, and other social determinants of poor mental health [42]. Group-based and interactive approaches positively affect diverse health concerns in a range of settings, including safe pregnancy and delivery, condom use and violence prevention [43–45]. Self-help groups can be delivered relatively cheaply, are flexible and adaptable, and due to their peer-oriented nature, give participants a stake in the group and its progress that our findings have shown is highly valued.

Given the short time frame of the pilot phase, this study did not assess longer-term outcomes nor monitor whether SHG could be sustained over time. Recruitment of AGYW proved challenging, partly a result of AGYW’s reluctance to engage with programmes due to pervasive social stigma. Future interventions would benefit from strategies to increase uptake and retention and develop ways to engage participants over a longer time period to move from building support networks to leveraging these for meaningful change. Nonetheless, the intervention did help AGYW overcome fears of attending health services as most attended a *Sisters* clinic by the end of the intervention.

Another strength of this study is that it tracked AGYW’s changing perceptions and experiences over the course of a participatory intervention that had been designed with stakeholder involvement of AGYW themselves. The active participation of adolescents in the formulation and implementation of interventions targeting them helps to ensure the acceptability and effectiveness of the intervention [46,47]. The study was able to gain access to close to 100 women from a very marginalised population, building on existing trust between the Sisters programme and community members [22,28,29].

A key limitation is the lack of a rigorous evaluation using a comparison group. The funding for this study supported a short pilot to test feasibility and acceptability, thus the observed changes in mental health and well-being could not be attributed to participation in SHG. Furthermore, participants may have provided socially desirable responses in baseline and endline surveys. To ensure eligibility for the intervention, AGYW gave responses they knew were required for enrolment, including falsifying age and pregnancy status. Additional bias could have affected the CMD screening tool. During in-depth interviews, AGYW may have over-emphasised positive feedback about the SHG. To minimise this, however, the researcher who conducted interviews had not been part of intervention delivery.

## Conclusions

Mental health, as a critical foundation for overall wellbeing and quality of life, is a growing priority for interventions targeting adolescents and young people. Those who are especially vulnerable, such as AGYW who sell sex, while pregnant or mothers to young children, experience high levels of stress and anxiety, thus confronting numerous risks to their mental health, yet there are few programmes tailored to their needs. The *Building Resilience* SHG intervention in Zimbabwe provides one practical intervention model that shows promise in mitigating this group’s poor mental health. Over six months, participating in an interactive SHG reduced participants’ feelings of isolation, offered knowledge and skills they valued, and appeared to lessen the experience of common mental health disorders. Although it requires more rigorous testing, the programme provides a foundation that can be built in a wide range of community settings.

## Paper context

Intersecting vulnerabilities experienced by adolescents, such as poverty, early parenthood, and involvement in transacting sex, lead to stress and anxiety. Few mental health interventions address the needs of adolescents confronting multiple challenges in resource poor settings. Participatory self-help groups tailored for marginalised adolescent mothers in Zimbabwe provided a new source of social support that led to collective agency. Peer-based groups are cost-effective, adaptable to different contexts, and have the potential to reduce common mental health disorders.
